# Cyclotron-based production of ^68^Ga, [^68^Ga]GaCl_3_, and [^68^Ga]Ga-PSMA-11 from a liquid target

**DOI:** 10.1186/s41181-020-00106-9

**Published:** 2020-11-12

**Authors:** Melissa E. Rodnick, Carina Sollert, Daniela Stark, Mara Clark, Andrew Katsifis, Brian G. Hockley, D. Christian Parr, Jens Frigell, Bradford D. Henderson, Monica Abghari-Gerst, Morand R. Piert, Michael J. Fulham, Stefan Eberl, Katherine Gagnon, Peter J. H. Scott

**Affiliations:** 1 Division of Nuclear Medicine, Department of Radiology, University of Michigan, Ann Arbor, MI USA; 2 GE Healthcare, GEMS PET Systems, Uppsala, Sweden; 3 Department of Molecular Imaging, Royal Prince Alfred Hospital, Sydney, Australia; 4 Department of Molecular Imaging, Royal Prince Alfred Hospital and Sydney Medical School, University of Sydney, Sydney, Australia; 5 Department of Molecular Imaging, Royal Prince Alfred Hospital and School of Computer Science, The University of Sydney, Sydney, Australia

**Keywords:** Gallium-68, Cyclotron targetry, Positron emission tomography, PSMA

## Abstract

**Purpose:**

To optimize the direct production of ^68^Ga on a cyclotron, via the ^68^Zn(p,n)^68^Ga reaction using a liquid cyclotron target. We Investigated the yield of cyclotron-produced ^68^Ga, extraction of [^68^Ga]GaCl_3_ and subsequent [^68^Ga]Ga-PSMA-11 labeling using an automated synthesis module.

**Methods:**

Irradiations of a 1.0 M solution of [^68^Zn]Zn(NO_3_)_2_ in dilute (0.2–0.3 M) HNO_3_ were conducted using GE PETtrace cyclotrons and GE ^68^Ga liquid targets. The proton beam energy was degraded to a nominal 14.3 MeV to minimize the co-production of ^67^Ga through the ^68^Zn(p,2n)^67^Ga reaction without unduly compromising ^68^Ga yields. We also evaluated the effects of varying beam times (50–75 min) and beam currents (27–40 μA). Crude ^68^Ga production was measured. The extraction of [^68^Ga]GaCl_3_ was performed using a 2 column solid phase method on the GE FASTlab Developer platform. Extracted [^68^Ga]GaCl_3_ was used to label [^68^Ga]Ga-PSMA-11 that was intended for clinical use.

**Results:**

The decay corrected yield of ^68^Ga at EOB was typically > 3.7 GBq (100 mCi) for a 60 min beam, with irradiations of [^68^Zn]Zn(NO_3_)_2_ at 0.3 M HNO_3._ Target/chemistry performance was more consistent when compared with 0.2 M HNO_3_. Radionuclidic purity of ^68^Ga was typically > 99.8% at EOB and met the requirements specified in the European Pharmacopoeia (< 2% combined ^66/67^Ga) for a practical clinical product shelf-life. The activity yield of [^68^Ga]GaCl_3_ was typically > 50% (~ 1.85 GBq, 50 mCi); yields improved as processes were optimized. Labeling yields for [^68^Ga]Ga-PSMA-11 were near quantitative (~ 1.67 GBq, 45 mCi) at EOS. Cyclotron produced [^68^Ga]Ga-PSMA-11 underwent full quality control, stability and sterility testing, and was implemented for human use at the University of Michigan as an Investigational New Drug through the US FDA and also at the Royal Prince Alfred Hospital (RPA).

**Conclusion:**

Direct cyclotron irradiation of a liquid target provides clinically relevant quantities of [^68^Ga]Ga-PSMA-11 and is a viable alternative to traditional ^68^Ge/^68^Ga generators.

## Introduction

**Fig. 1:**
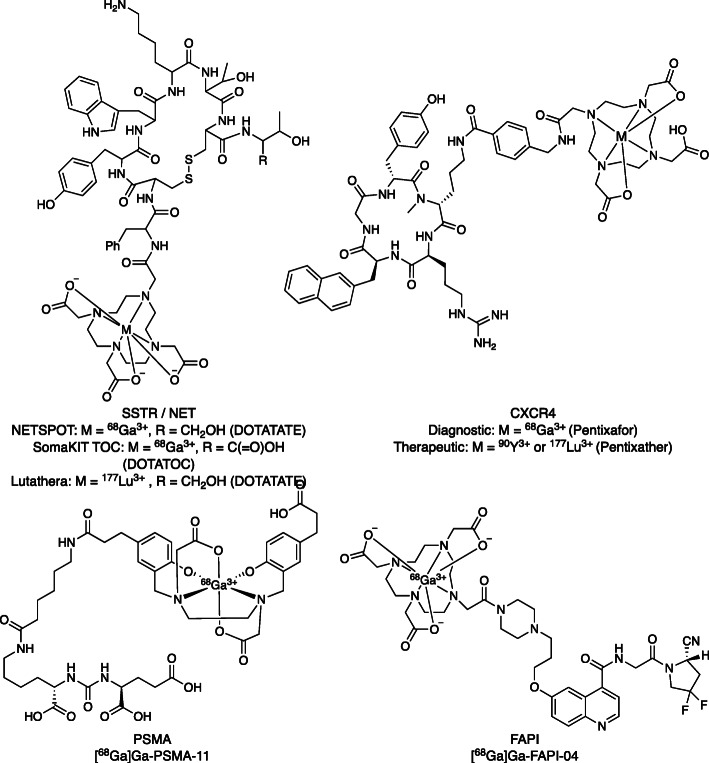
Theranostic Radiopharmaceuticals

The medicinal use of ^68^Ga was first described over 4 decades ago albeit with a very small clinical footprint for much of that time (Eder et al. 2014; Graham et al. 2017; Lenzo et al. 2018; Velikyan 2018). Over the past 15 years, there has been a surge in ^68^Ga radiopharmaceutical development, exceeding that of other radiotracers, with a 100-fold increase in the number of ^68^Ga publications. Over the last decade, there has also been a marked increase in the clinical use of ^68^Ga radiotracers that has been attributed to the ease of acquiring ^68^Ga from ^68^Ge/^68^Ga generators and the development and approval of new theranostic tracers (see Fig. 1 for some recent examples) (Baum and Kulkarni 2012). The diagnostic applications of ^68^Ga vary across jurisdictions/countries, but initial development was driven by development of theranostic agents targeting somatostatin receptors (SSTRs) for the PET imaging (^68^Ga, ^64^Cu) and either alpha (^213^Bi, ^225^Ac) or beta (^90^Y, ^177^Lu) therapy of neuroendocrine tumors (NETs) (Graham et al. 2017; Jackson et al. 2017). In the United States, DOTA-TATE labeled with ^68^Ga (NETSPOT) and ^177^Lu (Lutathera) are approved by the U.S. Food and Drug Administration for NET diagnosis and therapy, while the European Union approved [^68^Ga]Ga-DOTA-TOC (SomaKIT TOC) and Lutathera. Subsequent development of theranostic agents for infection/inflammation (Velikyan 2018), prostate cancer (Eder et al. 2014; Lenzo et al. 2018; Ruangma et al. 2018), C-X-C chemokine receptor type 4 (CXCR4) (Gourni et al. 2011; Herrmann et al. 2016) and, most recently, fibroblast activation protein inhibitors (FAPI) (Kratochwil et al. 2019) is further driving demand and highlights the need for access to a reliable (and economical) supply of ^68^Ga8 that is the focus of this paper. Analogous development of a reliable pipeline of therapeutic radionuclides is also an urgent need for the nuclear medicine community (Herrmann et al. 2020), but beyond the scope of this article.

Gallium-68 is usually eluted from a ^68^Ge/^68^Ga generator, and thus can be readily implemented in PET facilities that do not own a cyclotron. There are also many additional attributes of ^68^Ga that make it a desirable PET radionuclide and the first widely available PET radioactive metal ion (radiometal) for routine use globally. ^68^Ga is a positron emitting (89% β+) radionuclide with a 68 min half-life that, although relatively short, is compatible with distribution. The ^68^Ga^3+^ cation is small with an ionic radius of 0.62 Å, which behaves as a relatively hard Lewis acid with an affinity for binding ligands containing oxygen and nitrogen donors, and is suitable for conjugation to various biomolecular vectors using bifunctional chelators and various macromolecules including small molecules with rapid pharmacokinetic profiles, such as peptides and peptidomimetics (Blower et al. 2019; Martiniova et al. 2016; Smith et al. 2013). This synthetic diversity provides the ability for ^68^Ga kit development.

The most recent main contributor to the expansion of ^68^Ga-based PET has been imaging of the prostate specific membrane antigen (PSMA) with a host of new radiopharmaceuticals (Ruangma et al. 2018). Of these new agents, [^68^Ga]Ga-PSMA-11 has been most widely used to date (Hope et al. 2017). Prostate cancer is the second most common cancer found in men in the United States and the second most prevalent cause of cancer death in men (Prostate Cancer: Statistics 2020). Survival rates depend on the type of prostate cancer and the stage at diagnosis. Men with localized disease have a 5-year survival rate of nearly 100%. However, 20–40% of these patients develop biochemical recurrence (BCR) and the recurrent disease can be loco-regional or more widespread. Patients with metastatic disease have a markedly decreased 5-year survival rate of 30% (Prostate Cancer: Statistics 2020). The early and accurate identification of tumor recurrence and metastatic disease is essential for optimal patient management, but this remains a major challenge for traditional imaging methods with anatomical imaging and bone scintigraphy.

The imaging of PSMA expression with [^68^Ga]Ga-PSMA-11 and PET/CT has proven to be a highly effective and sensitive tool for patient management (Blower et al. 2019). While the primary use of [^68^Ga]Ga-PSMA-11 has been for detecting recurrent disease, it has also been successful at staging primary prostate cancer, and useful for guiding biopsies to improve sample accuracy, guiding surgery, and monitoring treatment response (Lenzo et al. 2018). There has been a positive clinical impact of [^68^Ga]Ga-PSMA-11 at the University of Michigan with a change in patient care management in 72% of the scanned patient population. A similar high impact has been reported in a large Australian study that cited a 51% change in care management (62% for BCR patients and 21% for primary staging) (Roach et al. 2018). In 2016 a study from Belgium reported that in patients who underwent a [^68^Ga]Ga-PSMA-11 scan there was a 76% impact in patient care management (Albisinni et al. 2017). A 2017 study from the University of California San Francisco reported a 53% change in patient management (Hope et al. 2017). Additionally, [^68^Ga]Ga-PSMA-11 has been used theranostically in conjunction with complementary ^177^Lu (i.e. β-) or ^225^Ac (i.e. α) therapeutic PSMA targeting agents. Such PSMA targeted therapies are currently undergoing evaluation in clinical trials in patients with castrate-resistant metastatic prostate cancer (Fendler et al. 2017; Kratochwil et al. 2018).

Since [^68^Ga]Ga-PSMA-11 has higher accuracy and sensitivity in detecting metastatic disease than [^18^F]fluorocholine, [^11^C]choline, and CT (Afshar-Oromieh et al. 2014; Blower et al. 2019; McCormick et al. 2019; Schwenck et al. 2017), a superior detection rate to [^18^F]fluciclovine (Calais et al. 2018), and overall superior clinical performance (Lenzo et al. 2018; McCormick et al. 2019; Schwenck et al. 2017), it is rapidly becoming the most commonly used radiotracer for prostate cancer management. This presents challenges in meeting the expected demand for the agent once regulatory approval is gained. To put this into context, [^11^C]choline has been one of the most widely used prostate cancer radiotracers in the US since its FDA approval in 2012 (Evans et al. 2018), and the busiest cancer centers reportedly perform 10–15 [^11^C]choline scans daily (Lowe and Kwon 2015). It has been possible to service this volume of patients given high yielding [^11^C]choline syntheses (> 7.4 GBq (200 mCi) /batch) (Shao et al. 2011; Shao et al. 2010), coupled with the ability to run production multiple times per day (limited only by cyclotron and synthesis module availability). Contrastingly, transferring such a patient population to exclusively [^68^Ga]Ga-PSMA-11 PET is not feasible in a workflow relying entirely on ^68^Ge/^68^Ga generators. While ^68^Ge/^68^Ga generators offer workflow simplicity for tracer production there are a number of limitations: a) current GMP generators have a maximum activity of 1.85 GBq (50 mCi) and are restricted to elutions every 3–4-h increments, which in practice typically means 2 production runs per day with 2–4 doses per day; b) two or more generators increase the number of patients doses to 6 or more, but still less than the requirements of busy cancer centers; c) commercial supply has not kept pace with the clinical demand and lead times for generator delivery can be up to 18 months in some markets (Cutler and Minoshima 2018); d) the eluted activity constantly declines over time and so to ensure a regular clinical supply of [^68^Ga]Ga-PSMA-11, multiple sequential and overlapping generators must be purchased throughout the year and; e) there is the potential for long lived parent ^68^Ge contamination and/or breakthrough. To this end, an additional source of ^68^Ga needs to be explored and implemented into the clinical setting to meet the current and future patient demand (Cutler and Minoshima 2018).

An attractive alternative to diversifying the supply of ^68^Ga is the direct production of ^68^Ga on a cyclotron, via the ^68^Zn(p,n)^68^Ga reaction. This alternative approach has garnered significant interest by the community, including publication of a European Pharmacopeia monograph for the direct accelerator-based production of [^68^Ga]GaCl_3_ which was published in draft form in 2018 and finalized in 2020 (Gallium (^68^Ga) chloride (accelerator-produced) solution for radiolabeling 2020) and a technical document published by the IAEA in support of direct production of ^68^Ga via liquid and solid targets (International Atomic Energy Agency 2019). There are two strategies for producing ^68^Ga via the ^68^Zn(p,n)^68^Ga reaction on a cyclotron - namely, liquid (Alves et al. 2017; do Carmo et al. 2020; International Atomic Energy Agency 2019; Jensen and Clark 2011; Nair et al. 2017; Oehlke et al. 2015; Pandey et al. 2019; Pandey et al. 2014; Pandey and DeGrado 2019) and solid targets (Boschi et al. 2019; Engle et al. 2012; Lin et al. 2018; Sadeghi et al. 2009; Schweinsberg et al. 2019; Tolmachev and Lundqvist 1996; Zeisler et al. 2019). Liquid targets offer implementation simplicity for sites familiar with [^18^F]FDG production as they present a similar workflow to production of [^18^F]fluoride and are compatible with laboratory set-ups in existing PET radiopharmaceutical production centers. Solid targets, however, typically impose increased requirements on infrastructure and/or local site expertise but offer more than an order of magnitude higher ^68^Ga yields (e.g. several GBq/Ci) (Lin et al. 2018; Schweinsberg et al. 2019). Regardless of opting for liquid or solid targets an efficient means for purifying the ^68^Ga from the irradiated ^68^Zn is required. The limitations of cyclotron produced ^68^Ga are obviously: a) a cyclotron with suitable targets, b) the co-production of ^67^Ga and ^66^Ga and, c) the potential for residual levels of ^68^Zn and other metal impurities affecting labeling efficiencies. These factors place stringent demands on the proton energy, the target material and reagent quality, and finally ^68^Zn/^68^Ga separation methods.

We present results of the liquid target-based production of ^68^Ga on GE PETtrace cyclotrons, with focus on yield of ^68^Ga and extraction of [^68^Ga]GaCl_3_ using the GE FASTlab Developer platform. Furthermore, to demonstrate the clinical relevance of this direct production method, a single FASTlab cassette was used to perform the ^68^Zn/^68^Ga purification and subsequent labeling of [^68^Ga]Ga-PSMA-11. The cyclotron produced [^68^Ga]Ga-PSMA-11 underwent full quality control, stability and sterility testing, and has been used in humans at the University of Michigan (UM, Ann Arbor Mi, USA) under the FDA’s Investigational New Drug (IND) program, and at Royal Prince Alfred Hospital (RPA, Sydney, Australia) under exemption of the Therapeutic Goods Act (TGA) in a TGA GMP-licensed facility. The results from UM, GEMS (GE Healthcare Uppsala, Sweden) and RPA are presented.

## Materials and methods

### Liquid target irradiations

**Fig. 2:**
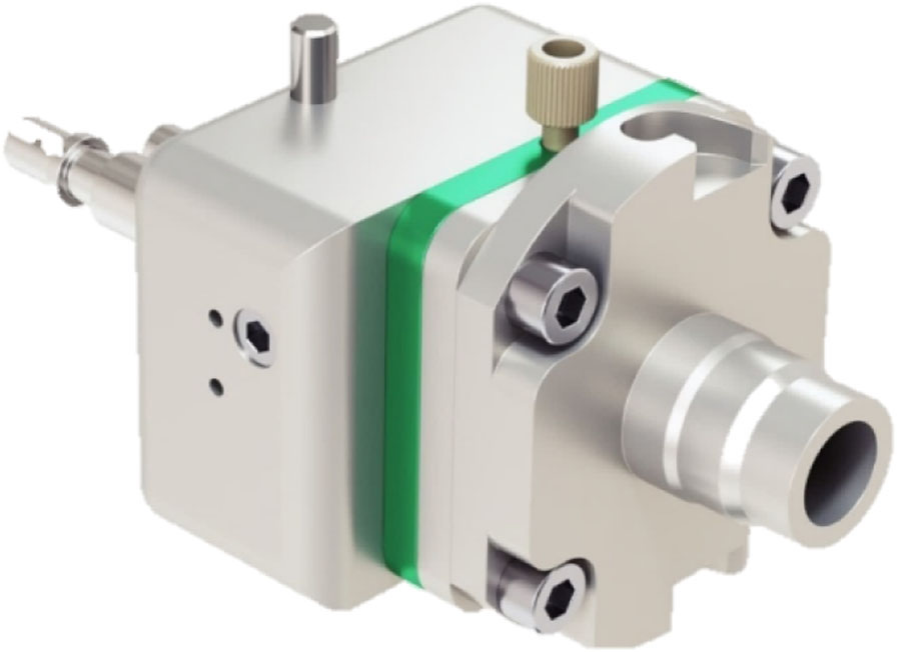
GE Gallium-68 Liquid Target

The GE ^68^Ga PETtrace Liquid Target (Fig. 2) is a water-cooled, gridded target without requiring He cooling of foils, designed specifically for ^68^Ga production. The target comprises a 200 μm thick aluminum energy degrader, a 25 μm Havar foil for support, and a 25 μm niobium foil for chemical inertness with the target media, thus rendering a nominal 14.3 MeV incident proton energy on the target media. Including the target lines/dead volume, the total target fill volume is approximately 2.2 mL.

The target media was prepared from isotopically enriched [^68^Zn]ZnO (Isoflex, USA) with addition of water (Ultrapur or 18 MΩ-cm) and 70% nitric acid (> 99.999% trace metal basis) to yield a 1.0 M solution of [^68^Zn]Zn(NO_3_)_2_ with an excess 0.2 M or 0.3 M HNO_3_ (both concentrations tested). All irradiations at UM and GEMS and the majority of irradiations at RPA employed the same lot of enriched ^68^Zn – namely: ^64^Zn (0.03%), ^66^Zn (0.16%), ^67^Zn (0.62%), ^68^Zn (99.16%), and ^70^Zn (0.03%), and from a chemical perspective, comprised 1 ppm iron. Recent irradiations at RPA used a different lot of enriched ^68^Zn – namely: ^64^Zn (0.1%), ^66^Zn (0.18%), ^67^Zn (0.96%), ^68^Zn (98.20%), and ^70^Zn (0.56%), and 3.1 ppm iron.

Irradiations were performed on GE PETtrace cyclotrons using the ^68^Ga Liquid Target and were typically 50–70 min in duration with beam currents of ~ 30–40 μA. Whenever possible, within the routine daily production schedule, a “cleaning” irradiation at 30–35 μA of typically 10–60 min was performed with dilute nitric acid (0.6 M) after irradiation of the [^68^Zn]Zn(NO_3_)_2_ solution.

### Chemical isolation on the FASTlab

#### Delivery to the FASTlab

**Fig. 3:**
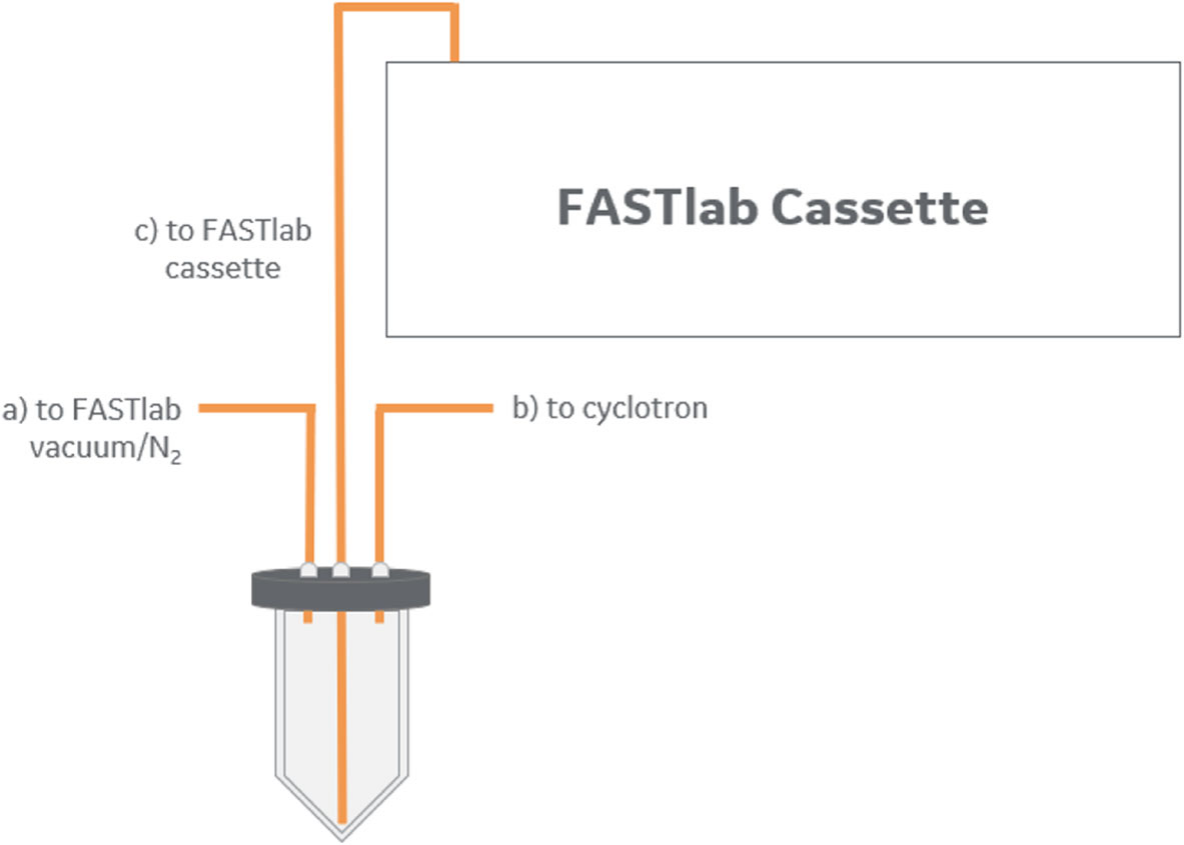
External vial for collection of the irradiated ^68^Zn solution

To facilitate use of the same FASTlab for both ^68^Ga and ^18^F processing and the dilution of the delivered ^68^Ga target solution, the irradiated target media was delivered from the cyclotron into an external 10 mL V-vial with connections to the FASTlab (Fig. 3). Thus, delivery of ^68^Ga target material completely bypasses the incoming activity plunger of the FASTlab module avoiding potential cross contamination between ^18^F and ^68^Ga target deliveries when the module is used for both types of targets. In this activity receiving vial, the ^68^Ga target solution was automatically diluted with water from the synthesis unit to achieve a nitric acid concentration of < 0.1 M required for subsequent processing. The diluted target solution is automatically loaded onto the cassette by nitrogen overpressure.

#### Chemical isolation of [^68^Ga]GaCl_3_

**Fig. 4:**
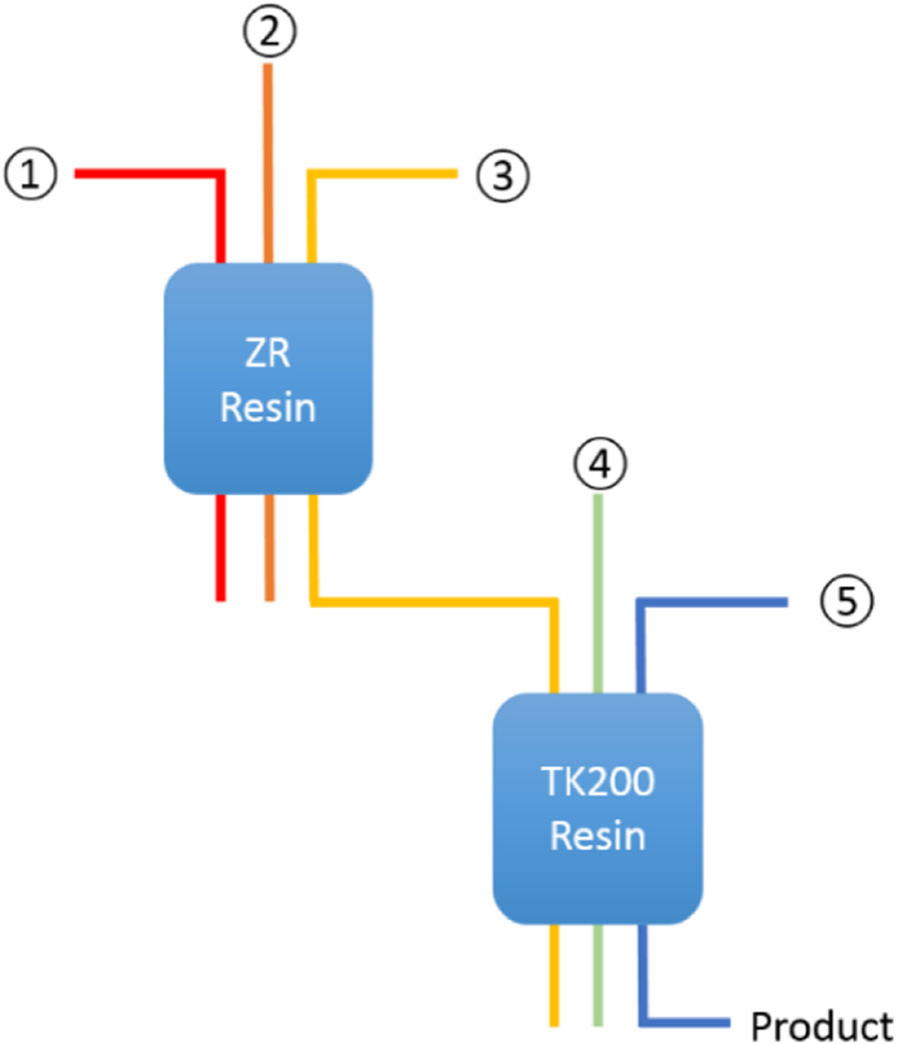
Two-column approach for ^68^Ga chemical separation

A primary goal of this effort was to develop a FASTlab cassette which allowed for [^68^Ga]GaCl_3_ extraction in a formulation comparable with existing generators. Additionally, on-line column conditioning, the use of minimum quantities of acid, and the exclusion of organic solvents or base-mediated pH adjustments were desired. Chemical isolation of [^68^Ga]GaCl_3_ was implemented on the GE FASTlab Developer platform. The process is based on the 2-column approach we have previously reported for liquid targets (Nair et al. 2017) and recently repeated by Riga and colleagues (Riga et al. 2018). The process is shown in Fig. 4. Initial separation of ^68^Ga from ^68^Zn is performed by trapping the ^68^Ga on a hydroxamate-based resin (ZR resin, Triskem) cartridge. Further purification, concentration and acid reduction is realized by using a TOPO-based resin (TK200 resin, Triskem) cartridge.

In our initial efforts, elution of the TK200 resin with water (Scheme A in Table 1) resulted in a [^68^Ga]GaCl_3_ solution containing approximately 0.6 M HCl due to residual HCl content in the cartridge. Implementation of a NaCl/HCl rinse (Mueller et al. 2012) for reduction of residual acid achieved a final [^68^Ga]GaCl_3_ formulation of 0.1 M HCl in 5 mL (Scheme B in Table 1). This formulation is directly comparable to commercially available ^68^Ge/^68^Ga generators and is compatible with formulations required for pharmaceutical cold kit labeling.

**Table 1:** High level schemes of [^68^Ga]GaCl_3_ purifications

	Scheme A*	Scheme B
ZR Load	< 0.1 M HNO_3_	
ZR Wash	15 mL 0.1 M HNO_3_	
ZR Elution / Trapping on TK200	5–6 mL ~ 1.75 M HCl	
TK Wash	–	3.5 mL 2.0 M NaCl in 0.13 M HCl
TK Elution	H_2_O	1–2 mL H_2_O followed by dilute HCl to formulate

*Process as reported previously (Nair et al. 2017)

Process steps:

0. Condition ZR cartridge with 0.1 M HNO_3_ (7 mL) and TK200 resin with sterile water for injection (7 mL) followed by 1.75 M HCl (4 mL) prior to use.
1. Trapping of ^68^Ga on a hydroxamate-based resin (2 mL [~ 700 mg] ZR resin, Triskem)
2. Rinsing of the resin to remove residual zinc
3. Elution onto a TOPO-based resin (2 mL [~ 700 mg] TK200 resin, Triskem)
4. Wash to decrease residual acid content, and
5. Final elution with water and dilute hydrochloric acid, volumes of which can be varied, to yield [^68^Ga]GaCl_3_ in the desired formulation (e.g. 5 mL of 0.1 M HCl)

The process was optimized over time (with regards to flow rates, volumes, cassette rinsing, etc), thus not all runs were identical with regards to time lists on the FASTlab. Nevertheless, the chemical process can be categorized into two primary schemes (as noted in Table 1). Building on Scheme A, Scheme B includes a wash step of the TK200 resin in order to reduce the residual acid content in the [^68^Ga]GaCl_3_ eluate. The purification time is approximately 30 min.

In comparison to recent literature, this method requires less acid and does not involve organic solvents or base-mediated pH adjustments, which is highlighted in Table 2.

**Table 2:** Comparison of FASTlab [^68^Ga]GaCl_3_ purification vs. recent literature

Reference	HNO_3_^a^ [mmol]	HCl [mmol]	Organic solvents	Base-mediated pH adjustment?
This work	2.4	16	No	No
(Oehlke et al. 2015)	–	886	Yes (Methanol)	No
(Alves et al. 2018)	–	265	Yes (HBr/acetone)	No
(Pandey and DeGrado 2019)	0.25	38	Yes (Acetonitrile)	Yes

^a^Does not account for HNO_3_ in the liquid target

**Fig. 5:**
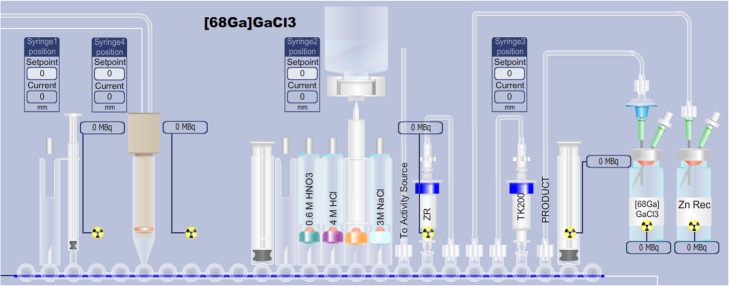
FASTlab cassette layout – applicable to Schemes “A&B” of Table 1

**Fig. 6:**
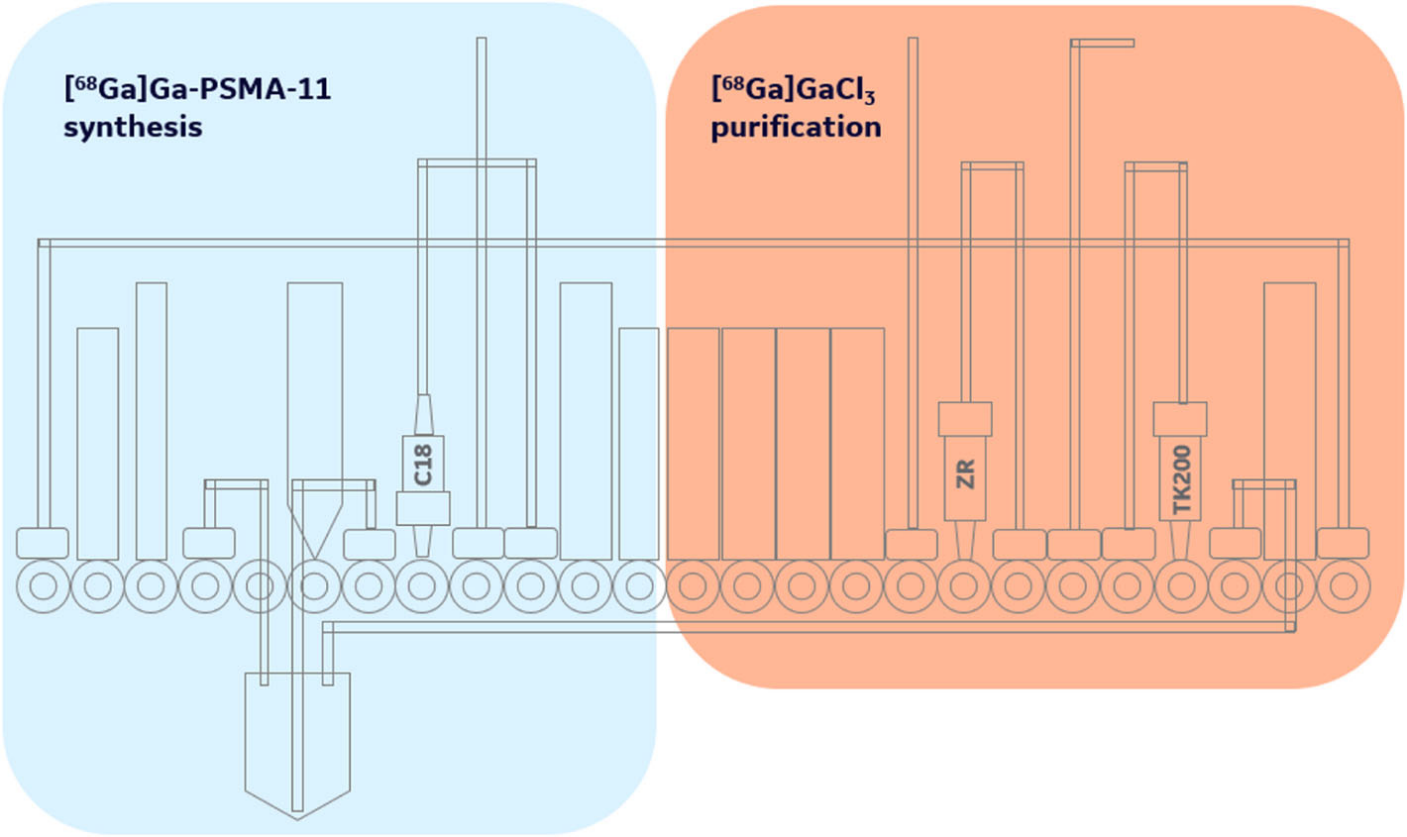
Partitioning of FASTlab cassette: Right-hand side is reserved for [^68^Ga]GaCl_3_ purification, and the left-hand side accommodates the [^68^Ga]Ga-PSMA-11 synthesis and C18 cartridge based purification

The cassette layout for the automated [^68^Ga]GaCl_3_ separation on the FASTlab is given in Fig. 5, noting that the [^68^Ga]GaCl_3_ chemistry is reserved to the right-hand side of the cassette. The left-hand side was kept vacant to enable subsequent on-cassette labeling (e.g. PSMA, NET tracers, etc), including C18 cartridge purification (Fig. 6). The line labeled “to activity source” is connected to the activity receiving vial (Fig. 3). Where applicable during the process, the vials of 0.6 M HNO_3_, 4 M HCl, and 3 M NaCl were automatically diluted and/or mixed to the desired concentrations by the FASTlab.

In advance of receipt of the activity the columns were automatically conditioned on the FASTlab, the ZR resin was conditioned with 0.1 M HNO_3_ (7 mL) and the TK200 was conditioned with both water (7 mL) followed by 1.75 M HCl (4 mL). Recycling of ^68^Zn is not presently being performed given the current availability and cost of ^68^Zn (approximately US$100 per target fill), however, the ^68^Zn solution is collected separately to facilitate future recycling.

#### Synthesis of [^68^Ga]Ga-PSMA-11

The direct cyclotron-based production of [^68^Ga]Ga-PSMA-11 was executed at the UM, GEMS and RPA using a single FASTlab cassette in a continuous process to perform both the [^68^Ga]GaCl_3_ isolation chemistry and subsequent PSMA-11 labeling, including C18 purification. Cassettes were prepared at each institution based on the FASTlab developer cassettes and accessories.

Initial labeling tests (UM) employed [^68^Ga]GaCl_3_ separation scheme “A”, with 10 μg PSMA-11 precursor in 1.5 M Hepes (1 mL) and 3 M NaOAc (1.3 mL) buffer. Scheme “B” developed and implemented at GEMS and RPA used 10 μg PSMA-11 precursor in 1.5 mL/1.0 M NaOAc (GEMS) or 1.3 mL/1.5 M NaOAc buffer (RPA) adjusted to pH 4.5–4.8. Approximately 3–4 mg L-ascorbic acid was also added (GEMS/RPA) to the precursor vial to minimize radiolysis during synthesis. An additional 20–21 mg of L-ascorbic acid (0.44 mL; 0.25 M) is also added directly into the product line at RPA as stabilizer of the final product. Labeling occurred for 5 min at 50 °C. At UM and RPA, the final product was reformulated into Phosphate Buffered Saline (PBS) using a C18 cartridge (preconditioned with 1.2 mL EtOH followed by 4.5 mL sterile water for injection). Briefly, at UM the crude reaction mixture was passed through the C18 cartridge to trap [^68^Ga]Ga-PSMA-11. The reaction vessel was rinsed with water (2.4 mL) and the rinse passed over the C18 cartridge. [^68^Ga]Ga-PSMA-11 was eluted from the C18 cartridge with EtOH/H_2_O (1:1 v:v, 2 mL), sterile filtered (Millipore Cathivex-GV, 0.22 μm) and diluted with PBS solution (14.5 mL) to give the final formulated product that was submitted for quality control testing.

#### Quality control of [^68^Ga]Ga-PSMA-11

Quality control testing of [^68^Ga]Ga-PSMA-11 doses was conducted according to the guidelines outlined in the U.S. and European Pharmacopeias using standard methods (visual inspection, pH, radionuclidic identity, sterile filter integrity, bacterial endotoxin analysis, and sterility testing) previously described (Mossine et al. 2020), as well as procedures specific for [^68^Ga]Ga-PSMA-11 and described below.

##### Representative TLC analysis of [^68^Ga]Ga-PSMA-11

At UM, radiochemical purity was determined by thin layer chromatography (TLC) using silica-backed 7.5 cm glass or paper chromatography plates and an Eckert and Ziegler AR-2000 TLC scanner. The plate was spotted with a sample of [^68^Ga]Ga-PSMA-11 product. The developing solution (mobile phase), 77 g/L ammonium acetate in a 50/50 water/methanol. The plate was developed in this solvent system, dried using a warm laboratory hotplate, and placed on the TLC scanner to determine radiochemical purity, which must be ≥90%.

##### Representative radio-HPLC analysis of [^68^Ga]Ga-PSMA-11

At UM, chemical and radiochemical purities/identities were analyzed using a Shimadzu LC2010 HPLC equipped with a radioactivity detector and an ultraviolet (UV) detector (column: Phenomenex C18(2) 250 × 4.6 mm column; mobile phase A: 0.1% trifluoroacetic acid in MeCN; mobile phase B: 0.1% trifluoroacetic acid in milli-Q water; Gradient: 0 min A:B = 5:95–10 min A:B = 30:70–11 min A:B = 30:70–11.1 min A:B = 5:95–15 min A:B = 5:95; flow rate: 1.0 mL/min; UV: 205 nm). On this QC HPLC system, [^68^Ga]Ga-PSMA-11 has a retention time of ~ 11 min. Radiochemical purity for doses was confirmed to be > 90%, and identity was confirmed by comparing the retention time of the radiolabelled product with that of the corresponding unlabelled reference standard (40 μg/mL).

##### Representative Gallium-66 and Gallium-67 concentration (Radionuclidic purity)

At UM, cyclotron-produced [^68^Ga]Ga-PSMA-11 was tested for ^66^Ga and ^67^Ga weekly to determine the fitness of the target produced ^68^Ga for use in production of clinical doses of [^68^Ga]Ga-PSMA-11 for the following week. A small sample of [^68^Ga]Ga-PSMA-11 was assayed via a dose calibrator to determine an initial radioactivity profile. The sample was then stored in a shielded environment for 24–72 h to allow ^68^Ga to decay to near-zero, and the sample analyzed by gamma spectroscopy. Decay-corrected values for the amount of ^66^Ga and ^67^Ga were determined and used in conjunction with the initial radioactivity profile value to determine the relative amount of both radionuclides in the final product. The recent EU Monograph on cyclotron-produced requires ≤2% combined ^66^Ga and ^67^Ga in doses until the end of the shelf-life.

## Results and discussion

### ^68^Ga yields

Total ^68^Ga yields from the target were assessed by: (a) downloading the total irradiated target contents into a vial placed in a dose calibrator without chemical purification and ensuring suitable decay time (90–120 min) or curve fitting to avoid any ^13^N contribution, or (b) measurement of residual activity of cassette components and product post [^68^Ga]GaCl_3_ isolation or post [^68^Ga]Ga-PSMA-11 labeling chemistry. For the data presented at GEMS, this includes an early series of 9 consecutive 60-min irradiations from 30 to 40 μA (entire target contents), and 20 consecutive irradiations (post-chemistry) following a target rebuild.

Radioactivity yields exceeding 100 mCi (3.7 GBq) at EOB are typical (see Table 3) with irradiation of [^68^Zn]Zn(NO_3_)_2_ at 0.3 M HNO_3_ yielding more consistent target/chemistry performance. Albeit higher acid concentrations have been reported in the literature (Pandey et al. 2019), we opted to maintain the excess nitric acid as low as possible to minimize corrosive wear on components and facilitate the subsequent chemistry (which requires < 0.1 M HNO_3_ for ZR resin loading).

**Table 3:** Summary of ^68^Ga productions and total ^68^Ga radioactivity yield at EOB

Site	HNO_3_ [M]	N	I [uA]	Beam time [min]	EOB activity [GBq]	EOB activity [mCi]	Measurement
UM	0.2	13	30	60	4.1 ± 0.6	112 ± 16	Entire target contents
	0.2	6	35	60	3.9 ± 0.6	106 ± 17	Entire target contents
	0.2	6	40	60	3.8 ± 0.4	102 ± 11	Entire target contents
	0.3	12	34 ± 4	60	4.6 ± 0.4	126 ± 12	Entire target contents
GEMS	0.2	9	36 ± 5	60	4.5 ± 0.3	120 ± 9	Entire target contents
	0.2	14	30	69 ± 7	3.5 ± 0.9	94 ± 24	Ʃ of parts post chemistry
	0.3	6	29 ± 1	70 ± 13	4.3 ± 0.5	115 ± 14	Ʃ of parts post chemistry
RPA	0.3	25	36 ± 2.2	60	4.0 ± 0.6	107 ± 17	Entire target contents
	0.3	53	35	60	3.8 ± 0.5	104 ± 14	Ʃ of parts post chemistry

While it is theoretically possible to increase the target yields by increasing the ^68^Zn concentration, the 1.0 M solution used here facilitates transfer to the hot cell (i.e. the solution is not too viscous). Should multi-Ci yields of ^68^Ga be desired, adoption of the proposed method to solid targets as has been reported previously by taking advantage of ^68^Ga trapping on ZR resin in high HCl concentration loading conditions (Schweinsberg et al. 2019).

### [^68^Ga]GaCl_3_, [^68^Ga]Ga-PSMA-11 – yields and quality

Several hundred irradiations and purifications/labelings have been performed throughout the development efforts, however, for sake of brevity, we report herein on several representative subsets of experimental data. These data are summarized in Tables 4, 5 and 6.

**Table 4:** High-level summary of ^68^Ga runs reported herein for UM, GEMS and RPA

	Site	N	comment
[^68^Ga]GaCl_3_	UM	27	60 min beam current
	GEMS	13	Consecutive productions, 0.2 or 0.3 M HNO_3_
	RPA	20	60 min 35 μA beam, 0.3 M HNO_3_
[^68^Ga]Ga-PSMA-11	UM	3 + 35	Validation + clinical
	GEMS	3	Consecutive productions
	RPA	8	Validation + clinical

**Table 5:** Overview of [^68^Ga]GaCl_3_ productions (EOS)

Site	Chemistry Scheme	HNO_3_ [mol/L]	I [μA]	Beam time [min]	N	Product activity	
						[GBq]	[mCi]
UM	A	0.2	30	60	15	2.0 ± 0.3	54 ± 8
			35		6	2.0 ± 0.3	55 ± 8
			40		6	1.9 ± 0.2	50 ± 5
GEMS	B	0.2	30	64 ± 6	10	1.7 ± 0.5	46 ± 13
		0.3	29 ± 1	73 ± 6	3	2.5 ± 0.1	67 ± 3
RPA	B	0.3	35	60	20	2.0 ± 0.2	55 ± 6

**Table 6:** Overview of [^68^Ga]Ga-PSMA-11 productions (EOS)

Site	HNO_3_ [mol/L]	I [μA]	Beam time [min]	N	Product activity		Notes
					[GBq]	[mCi]	
UM	0.2	30–40	60	3	1.6 ± 0.3	43 ± 9	Validation runs
UM	0.2	30–40	60	35	1.7 ± 0.2	45 ± 6	Clinical
GEMS	0.3	30	64 ± 4	3	2.1 ± 0.4	57 ± 10	R&D efforts
RPA	0.3	35	60	14	1.6 ± 0.1	44 ± 3	Final validation and clinical (5) runs

Table 5 clearly demonstrates a robust routine production of ~ 1.85 GBq (~ 50 mCi) of [^68^Ga]GaCl_3_ via the liquid target cyclotron route. This compares favorably with approximately ~ 1.48 GBq (~ 40 mCi) of [^68^Ga]GaCl_3_ from a brand new, highest commercially available activity GMP generator with ~ 1.85 GBq (~ 50 mCi) of ^68^Ge. Furthermore, the eluted ^68^Ga activity steadily decreases over time due to the decay of the ^68^Ge.

[^68^Ga]Ga-PSMA-11 activity yields at EOS varied slightly across sites (Table 6) which may be at least partly attributed to beam parameters, state of target and slightly different labeling conditions used. At RPA, 3 patients can be readily scanned from a single batch of [^68^Ga]Ga-PSMA-11 using 2 scanners, which is the same number of patients which can be scanned with [^68^Ga]Ga-PSMA-11 produced from 2 staggered ^68^Ge/^68^Ga generators. As more than 2 productions runs can potentially be performed with the target, the number of patients able to be scanned per day is potentially increased.

Although activity yield is an important parameter to measure process performance, the quality of the cyclotron-produced [^68^Ga]GaCl_3_ is of even greater importance as high quality [^68^Ga]GaCl_3_ is critical to enable efficient labeling. Therefore, in addition to yield measurement and periodic quality control (QC) assessment, validation studies for [^68^Ga]GaCl_3_ and [^68^Ga]Ga-PSMA-11 were carried out at UM and for [^68^Ga]Ga-PSMA-11 at RPA. The test methods performed (half-life, radiochemical purity, pH, radionuclidic purity, metal analysis) were in accordance with the Ph. Eur. monograph for [^68^Ga]GaCl_3_ (Gallium (^68^Ga) chloride (accelerator-produced) solution for radiolabeling 2020) and are described in Section 2.2.4, with the exception of testing the Fe and Zn content, for which semi-quantitative colorimetric test strips (e.g. EM-Quant, Merck) and/or ICP-MS were used.

After we were confident with the performance of both the target and synthesis methods, we moved forward and conducted validation runs for both [^68^Ga]GaCl_3_ and [^68^Ga]Ga-PSMA-11. Table 7 reports the QC results for the [^68^Ga]GaCl_3_ validation runs carried out at UM, and all of the validation runs met or exceeded the established criteria in the European Pharmacopoeia (Gallium (^68^Ga) chloride (accelerator-produced) solution for radiolabeling 2020). Subsequently, validation of [^68^Ga]Ga-PSMA-11 was also undertaken. QC testing results for [^68^Ga]Ga-PSMA-11 are shown in Table 8 (UM) and Table 9 (RPA), with RCP assessment by radio-TLC and HPLC (see Section 2.2.4). Endotoxin, 4-h stability (data not shown), and sterility testing were also performed for the three validation runs. The 3 validation runs met (or exceeded) all QC criteria at end-of-synthesis and at the 4-h stability time point.

**Table 7:** Quality Control Data for three [^68^Ga]GaCl_3_ validation runs (UM)^a^

TEST	1	2	3	Avg & SD	Release Criteria (Ph. Eur.)
Radiochemical Purity [^68^Ga]GaCl_3_ (iTLC-SG)	99	98	98	98.3 ± 0.3	≥ 95
Rf [^68^Ga]GaCl_3_ (TLC)	< 0.2	< 0.2	< 0.2	< 0.2	≤ 0.2
Rf Ref B^b^ (TLC)	> 0.7	> 0.7	> 0.7	> 0.7	≥ 0.7
pH	< 2	< 2	< 2	< 2	< 2
Visual Inspection	Passed	Passed	Passed	N/A	Clear, colorless, no visible particulate
Radionuclidic Identity (t_½_)	67.2	68.8	69.1	68.4 ± 0.8	64.6–71.4 min
Endotoxin Analysis	< 2	< 2	< 2	< 2	≤ 58.3 EU/mL
Fe μg/GBq	< 5	< 5	< 5	< 5	≤ 10 μg/GBq
Zn μg/GBq	< 1.25	< 1.25	< 1.25	< 1.25	≤ 10 μg/GBq
RNP at EOB (MCA)	99.8	99.8	99.8	99.8	≥ 98% (at shelf-life)

^a^After FASTLab isolation; ^b^ Reference solution B (Pentetic acid solution) from the European Pharmacopoeia (Gallium (^68^Ga) chloride (accelerator-produced) solution for radiolabeling 2020)

**Table 8:** Quality Control Data for three [^68^Ga]Ga-PSMA-11 validation runs (UM)

Tests	1	2	3	Avg & SD	Release Criteria (UM)
Radiochemical Purity (via TLC)	99.5	99.4	99.3	99.4 ± 0.1	≥ 90%
Relative Retention time (via HPLC)	1.004	1.005	1.005	1.0046 ± 0.0003	RRT: 0.9–1.1
pH	7.0	7.0	7.0	7.0	4.0–8.0
Visual Inspection	Passed	Passed	Passed	N/A	Clear, colorless, no visible particulate
Radionuclidic Identity (t_½_)	67.61	68.45	67.20	67.75 ± 0.53	64.6–71.4 min
Endotoxin Analysis	< 2	< 2	< 2	< 2	≤ 10.9 EU/mL
Bubble Point (PSI)	51	52	53	52 ± 1	≥ 50 PSI
Sterility	Passed	Passed	Passed	Passed	Complies with USP< 71>^a^
RNP at EOB (MCA)	99.8	99.8	99.8	99.8	≥ 98% (at time of use)

^a^See: USP 71 Microbiological Tests/Sterility Tests 2012

**Table 9:** Quality Control Data for three [^68^Ga]Ga-PSMA-11 validation runs (RPA)

Tests	1	2	3	Avg & SD	Release Criteria (RPA)
Radiochemical Purity (via TLC)	99.94	99.99	99.94	99.96 ± 0.03	≥ 95%
Radiochemical Purity (via HPLC)	99.94	99.97	100.0	99.97 ± 0.03	≥ 95%
pH	5.0	5.5	5.5	5.3 ± 0.3	4.0–8.0
Visual Inspection	Passed	Passed	Passed	N/A	Clear, colorless, no visible particulate
Radionuclidic Identity (t_½_)	67.9	68.1	67.7	67.9 ± 0.20	62–74 min
Endotoxin Analysis	< 1	< 1	< 1	< 1	≤ 17.5 EU/mL
Bubble Point (bar)	4.1	4.2	4.1	4.1 ± 0.06	≥ 3.5 bar
Sterility	Passed	Passed	Passed	Passed	Sterile – no growth
RNP at EOS (Well Counter)	99.7	99.8	99.8	99.8 ± 0.06	≥ 98% (at time of use)

The validation data shown here demonstrates the high quality of cyclotron-produced [^68^Ga]GaCl_3_ and [^68^Ga]Ga-PSMA-11, and highlights the reliability and reproducibility of both processes. Notably, the reported RNP satisfied the proposed EU Pharmacopoeia limits (≤2% of combined ^66^Ga + ^67^Ga for the shelf life of the product) (Gallium (^68^Ga) chloride (accelerator-produced) solution for radiolabeling 2020). Setting this criteria for duration of shelf life and the varying amounts of ^66^Ga + ^67^Ga in each batch mean that the shelf-life will vary batch to batch. In this work the RNP was typically 0.2% at EOS. This allows determination of the shelf life to be approximately 3.5 h, after which time the amount of ^66^Ga + ^67^Ga will exceed 2% due to radioactive decay of shorter-lived ^68^Ga. The dosimetry of the 2% ^66^Ga + ^67^Ga limit has been previously reported using worst-case assumptions, such as no biological clearance and rapid organ uptake (Graves et al. 2018). For this scenario, a relative dose increase up to 20% is reported but is typically less than 10% when compared to “pure” ^68^Ga (i.e. not comparing with generator ^68^Ga which may contain ^68^Ge). Overall, the obtained results provided a solid basis for the clinical evaluation of cyclotron-produced [^68^Ga]Ga-PSMA-11.

### Clinical production and use of [^68^Ga]Ga-PSMA-11

The necessary FDA approval was obtained to translate cyclotron-based [^68^Ga]Ga-PSMA-11 into clinical use at UM and evaluation began in 2019. At RPA, cyclotron-based ^68^Ga used for clinical [^68^Ga]Ga-PSMA-11 production began in 2020. The final manufacturing process takes ~ 2 h from start of irradiation to release of the dose to the clinic. For example, the process at UM proceeds as follows:

- Cyclotron irradiation (60 min)
- [^68^Ga]Ga-PSMA-11 synthesis (35 min)
- Quality control testing (25 min)

**Fig. 7:**
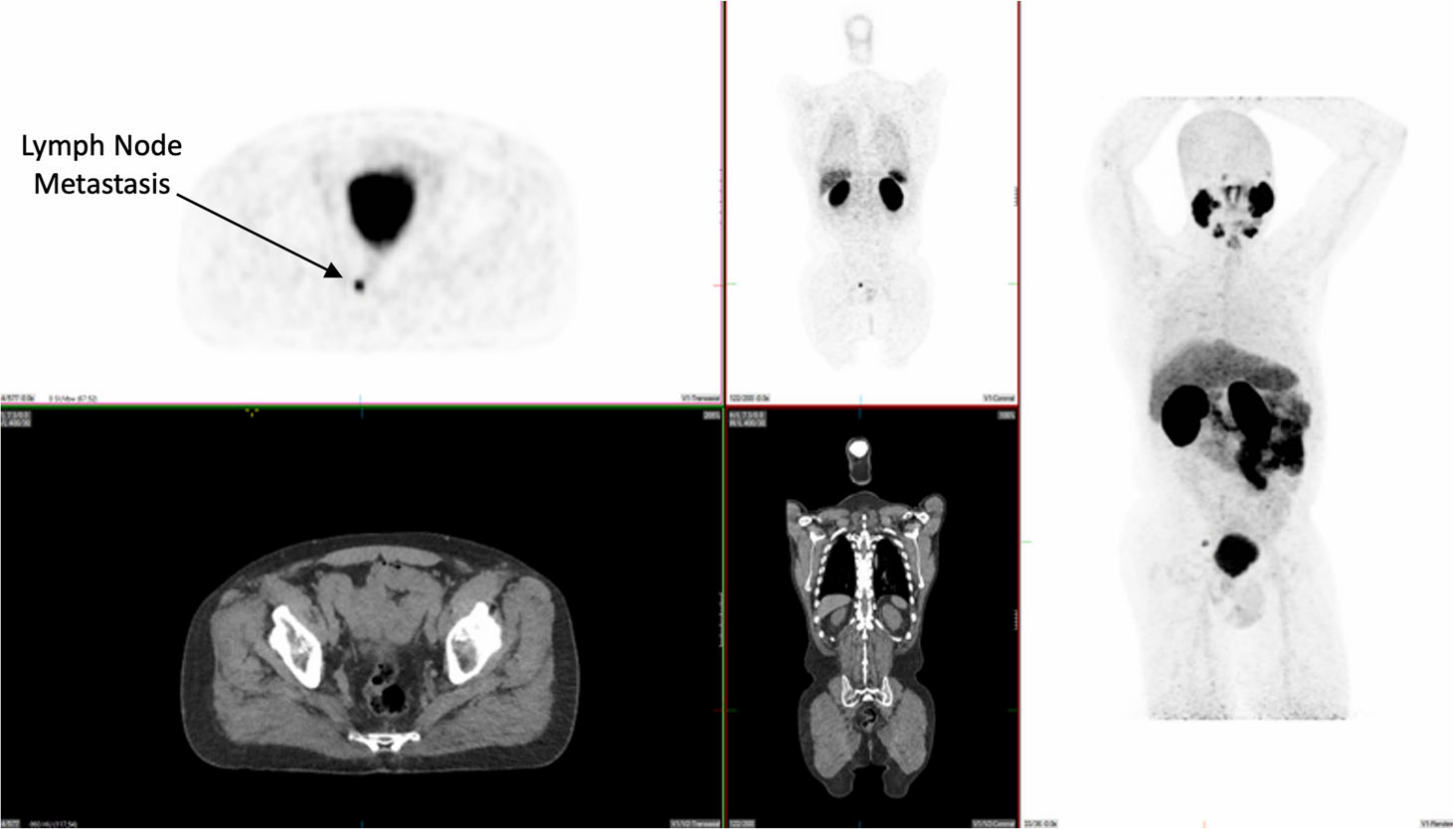
Images from the first patient scanned with [^68^Ga]Ga-PSMA-11 labeled with cyclotron produced ^68^Ga at the University of Michigan

To date, over 700 patients have been scanned with [^68^Ga]Ga-PSMA-11 at UM under our IND approval. Initially, this was with generator-based [^68^Ga]Ga-PSMA-11. We amended the IND to include cyclotron-based [^68^Ga]Ga-PSMA-11, and the first clinical production of cyclotron-based [^68^Ga]Ga-PSMA-11 from a single FASTlab cassette occurred in February 2019. As of March 2020, 50 clinical batches of cyclotron-produced [^68^Ga]Ga-PSMA-11 have been manufactured and used to scan more than 90 patients (see Table 6). A representative image from the first patient scanned with cyclotron-based [^68^Ga]Ga-PSMA-11 is shown in Fig. 7. We have noted no differences in the quality of studies where ^68^Ga was produced from a cyclotron when compared to studies using ^68^Ga obtained from a generator, and no pharmacological or physiological changes have been observed after intravenous administration of either generator-based or cyclotron-based [^68^Ga]Ga-PSMA-11.

## Conclusions and outlook

A process for isolating high purity [^68^Ga]GaCl_3_ from cyclotron-produced ^68^Ga and subsequent labeling of PSMA-11 on the GE FASTlab synthesizer with both steps being performed on a single cassette has been developed. The cyclotron-based method offers a reliable source of ^68^Ga and delivers consistently higher yields than currently available commercial 1.85 GBq (50 mCi) ^68^Ge/^68^Ga generators. Furthermore, in contrast to generators, for which ^68^Ga activity falls over time due to ^68^Ge decay, cyclotron-based ^68^Ga activity is consistent with time thereby simplifying patient scheduling. The FASTlab-derived [^68^Ga]GaCl_3_ solution for radiolabeling met the requirements in the European Pharmacopeia with the purity of reagents and ^68^Zn enrichment and purity used at these sites, and validation of [^68^Ga]GaCl_3_ and [^68^Ga]Ga-PSMA-11 for clinical application has been demonstrated by the UM and RPA. The total manufacturing time is approximately 2 h and over 90 patients have been scanned using cyclotron-based [^68^Ga]Ga-PSMA-11 to date. The process is in routine use to meet the growing demands for PSMA-based PET imaging at UM, and the first clinical studies have also been conducted at RPA. Additional studies to broaden the applicability of the [^68^Ga]GaCl_3_ process for labeling with other commonly used chelators such as DOTA have been performed successfully at RPA, and clinical use of [^68^Ga]Ga-DOTA-TATE labeled with cyclotron-produced ^68^Ga is ongoing. Similar studies are also currently ongoing at other sites.
